# Anti-Xa activity and hemorrhagic events under extracorporeal membrane oxygenation (ECMO): a multicenter cohort study

**DOI:** 10.1186/s13054-021-03554-0

**Published:** 2021-04-02

**Authors:** Richard Descamps, Mouhamed D. Moussa, Emmanuel Besnier, Marc-Olivier Fischer, Sébastien Preau, Fabienne Tamion, Cédric Daubin, Nicolas Cousin, André Vincentelli, Julien Goutay, Damien Du Cheyron

**Affiliations:** 1 Department of Medical Intensive Care, Caen University Hospital, 14000 Caen, France; 2 Inserm, CHU Lille, Surgical Critical Care, Department of Anesthesiology and Critical Care, Institut Pasteur de Lille, UMR1011-EGID, Univ. Lille, 59000 Lille, France; 3 Department of Anesthesiology and Critical Care, Rouen University Hospital, 76000 Rouen, France; 4 Department of Anesthesiology and Critical Care, Caen University Hospital, 14000 Caen, France; 5 Department of Medical Intensive Care, Lille University Hospital, 59000 Lille, France; 6 UNIROUEN, Inserm U1096, FHU- REMOD-VHF, Normandie Univ, 76000 Rouen, France; 7 Department of Medical Intensive Care, Rouen University Hospital, 76000 Rouen, France; 8 Inserm, CHU Lille, Department of Cardiac Surgery, Institut Pasteur de Lille, UMR1011-EGID, Univ. Lille, 59000 Lille, France

**Keywords:** Acute respiratory distress syndrome (ARDS), Extracorporeal membrane oxygenation (ECMO), Extracorporeal life support (ECLS), Hemorrhage, Anti-Xa

## Abstract

**Background:**

Hemorrhagic events remain a major concern in patients under extracorporeal membrane oxygenation (ECMO) support. We tested the association between anticoagulation levels and hemorrhagic events under ECMO using anti-Xa activity monitoring.

**Methods:**

We performed a retrospective multicenter cohort study in three ECMO centers. All adult patients treated with veno-venous (VV)- or veno-arterial (VA)-ECMO in 6 intensive care units between September 2017 and August 2019 were included. Anti-Xa activities were collected until a hemorrhagic event in the bleeding group and for the duration of ECMO in the non-bleeding group. All dosages were averaged to obtain means of anti-Xa activity for each patient, and patients were compared according to the occurrence or not of bleeding.

**Results:**

Among 367 patients assessed for eligibility, 121 were included. Thirty-five (29%) presented a hemorrhagic complication. In univariate analysis, anti-Xa activities were significantly higher in the bleeding group than in the non-bleeding group, both for the mean anti-Xa activity (0.38 [0.29–0.67] vs 0.33 [0.22–0.42] IU/mL; *p* = 0.01) and the maximal anti-Xa activity (0.83 [0.47–1.46] vs 0.66 [0.36–0.91] IU/mL; *p* = 0.05). In the Cox proportional hazard model, mean anti-Xa activity was associated with bleeding (*p* = 0.0001). By Kaplan–Meier analysis with the cutoff value at 0.46 IU/mL obtained by ROC curve analysis, the probability of survival under ECMO without bleeding was significantly lower when mean anti-Xa was > 0.46 IU/mL (*p* = 0.0006).

**Conclusion:**

In critically ill patients under ECMO, mean anti-Xa activity was an independent risk factor for hemorrhagic complications. Anticoagulation targets could be revised downward in both VV- and VA-ECMO.

**Supplementary Information:**

The online version contains supplementary material available at 10.1186/s13054-021-03554-0.

## Introduction

The use of extracorporeal membrane oxygenation (ECMO) is increasing in intensive care unit (ICU) settings, either for hemodynamic failure [veno-arterial (VA) ECMO] or for respiratory failure [veno-venous (VV) ECMO]. These techniques are used as rescue therapies in most cases and are associated with a high mortality rate, ranging from 31% for VV-ECMO to 59% for extracorporeal pulmonary resuscitation [1]. Hemorrhagic events under ECMO support remain a major concern and contribute to the mortality.

According to the extracorporeal life support organization (ELSO) register, there are 24% hemorrhagic complications under ECMO [2]. In the EOLIA randomized trial, focusing on severe ARDS patients, hemorrhagic complications leading to red blood cells (RBC) transfusion in the patients under VV-ECMO were 46%, versus 28% in the control group without ECMO [3], highlighting the high prevalence of these complications. In the LIFEGARDS international cohort including 350 patients with ARDS, authors reported hemorrhagic events in 25% of patients. These events were associated with a higher 6-month mortality [4]. Finally, in a recent meta-analysis including 21 studies (7190 patients under VV- or VA-ECMO), prevalence of hemorrhagic events was 17 to 51% [5].

These hemorrhagic complications are vectors of mortality [4, 6] and of blood products consumption, with 46 to 100% of the patients under ECMO support receiving transfusion [7]. Among these complications, intracerebral hemorrhage represents 2.2% to 4% of cases [7, 8]. Bleeding under ECMO is often iatrogenic with several favoring factors. Besides bleeding from the cannulation site, many hemostatic disorders are recognized as bleeding risk factors such as thrombocytopenia [5] and acquired von Willebrand syndrome [9–11]. In addition, the presence of an extracorporeal circulation device in human vessels requires preventive anticoagulation to avoid thromboembolic (TE) complications and clotting of the membrane, the centrifugal pump, or cannulas. Clot formation on the membrane oxygenator can lead to reduced gas exchange capacities or even necessity to change ECMO membrane and circuit, as previously described in a pediatric population [12]. A recent study also found an association between low doses of heparin and the number of oxygenator changes in VV ECMO [13].

Regarding ECMO specifically, guidelines support an effective anticoagulation regimen, with activated clotting time (ACT) levels between 180 and 250 [14]. Biological samples to guide anticoagulation must include ACT, activated partial thromboplastin time (aPTT) or anti-Xa activity [15], which seems to be more accurate in the ICU setting [16]. Targets used by most of ELSO centers are between 0.3 to 0.7 IU/mL for VA-ECMO [15], and targets recommended by some societies for VV-ECMO are 0.2 to 0.4 [17]. Despite these guidelines, anticoagulation targets strongly differ between medical teams and countries due to anchored practices. As an example, targets can range from 1.5 to 3 for the aPTT ratio, and from 0.2 to 1 for the anti-Xa activity for VV-ECMO, according to the results of a recent international survey [18]. Until today, only ACT and aPTT were identified as risk factors for hemorrhagic complications in ICU patients [8, 19, 20], while a growing number of medical teams now use anti-Xa activity as monitoring tool for anticoagulation in ECMO patients, with different targets. Only a recent study evaluated the association between maximum anti-Xa and hemorrhage, but the association was not significant [21]. Moreover, ICU patients have characteristics that make aPTT and ACT interpretation difficult, such as factor XII deficiency due to inflammation, antiphospholipid antibodies, heparin resistance, and as mentioned in a recent review by Chlebowski and colleagues, the anti-Xa assay correlates better with heparin concentration than the ACT or the aPTT [22].

In summary, heparinization may be a modifiable risk factor for hemorrhage and there is a need to standardize practices with well-defined targets according to the type of ECMO support. The objective of this study was to assess the association between anti-Xa levels and the occurrence of hemorrhagic complications for patients under VV- or VA-ECMO, then to identify a risk threshold.

## Method

### Study population

The *AntHem ECMO* (Anti-Xa and hemorrhage under ECMO) study was a retrospective multicenter cohort study, performed in six ICUs of three French academic hospitals (i.e., Caen University Hospital, Rouen University Hospital, and Lille University Hospital). All adult patients who had benefited from a VV- or VA-ECMO admitted in medical or surgical ICU between September 2017 and August 2019 were selected for inclusion. Cases with the absence of anti-Xa activity dosage during ECMO, moribund condition with death occurring during the first 24 h after ECMO implementation, hemorrhage before ECMO implementation, post-cardiotomy cardiogenic shock requiring ECLS, heparin-induced thrombocytopenia (HIT) occurring during ECMO, hemostatic disorders before ECMO, and lack of data regarding occurrence of hemorrhage, were excluded. Patients requiring therapeutic anticoagulation prior to ECMO implantation were not excluded.

### Ethics

The study protocol was declared and approved by Ethical Committee of the Caen University Hospital (Identification Number: 887). Informed consent was waived because of the retrospective nature of the analysis.

### Data collection

Patient information was obtained mainly through screening of Electronic Health Records and ECMO databases. Data collection was obtained from computerized and manuscript medical charts for Caen University Hospital, and only on computerized medical charts for the other centers. Baseline characteristics included gender, age, body mass index (BMI), the comorbid conditions such as active smoking and drinking, chronic obstructive pulmonary disease (COPD), asthma, hypertension, dilated cardiomyopathy, ischemic cardiomyopathy, chronic kidney disease (CKD), history of cancer, and long-term medications interfering with hemostasis. Severity of illness according to the sequential organ failure assessment (SOFA) score [23] and the Simplified Acute Physiology Score (SAPS II) [24] were also collected at the day of admission in ICU and at the day of ECMO cannulation. Glasgow coma scale was recorded just before ECMO cannulation. Biological data before ECMO cannulation such as hemoglobin level, platelet count, prothrombin ratio (PR) and creatininemia, as well as data of interest for adjustment during ECMO, including the highest values of creatininemia and bilirubinemia, and the lowest platelet count, were recorded. The administration and type of antiplatelet agents during ECMO was also collected. Finally, outcomes were assessed with 30-day mortality, hospital mortality, and ICU and hospital length of stays.

Regarding anticoagulation, unfractionated heparin was administered continuously to patients under ECMO in the three centers, with close anti-Xa monitoring, at least once daily and every 4 to 6 h after initiation or modification of heparin dosage, in two centers (Lille and Caen) and aPTT monitoring in one center (Rouen). Concerning the laboratory methods used to measure anti-Xa levels, a liquid chromogenic assay based on a synthetic chromogenic substrate and on Factor Xa inactivation was used in all centers with an automated analyzer (Analyzers and reagents detailed in the Additional file 1). Anti-Xa targets were personalized for each patient according to the type of ECMO and comorbidities, and varied among centers, ranging from 0.2 to 0.7 IU/mL. All anti-Xa activities were collected until occurrence of hemorrhage in the bleeding group, and for the duration of extracorporeal support in the non-bleeding group. All dosages were averaged to obtain mean anti-Xa activity for each patient. The highest value of anti-Xa activity was also recorded. Similarly, mean dose of heparin in IU/kg/h was calculated for each patient until hemorrhage for the bleeding group and until the end of ECMO for the non-bleeding group. Data such as search for acquired von Willebrand syndrome, vWF and tranexamic acid administration were not collected because therapeutic attitude varied among centers.

We also collected more specific data regarding intracerebral hemorrhages, delays between cannulation and occurrence of hemorrhage, number of packed RBC transfused during extracorporeal support, and occurrence of thrombo-embolic (TE) complications. Protocols concerning blood products transfusion were different between centers, with hemoglobin thresholds usually above 7 to 8 g/dL in the absence of hemorrhage.

### Definitions

Hemorrhagic complication was defined in accordance with the ELSO definition of serious bleeding [15]: clinically overt bleeding requiring administration of 2 packed RBC or more in less than 24 h or a drop in the hemoglobin of at least 2 g/dL in a 24-h period (except for hemolysis) and/or a bleeding characterized by its location (hemothorax, central nervous system, retroperitoneal hemorrhage) and/or a bleeding requiring specific intervention (embolization, surgery). Major hemorrhage was defined by a hemorrhage as mentioned above leading to death or therapeutic limitation and/or a complication as mentioned above requiring transport of the patient for a surgery or a hemostatic procedure and/or a massive transfusion requirement (more than half of the blood mass in less than 24 h).

### Objective

The primary outcome of the study was the association between mean anti-Xa activity under extracorporeal support and occurrence of any hemorrhagic complication, in order to determine a risk threshold.

The secondary outcomes were the association between occurrence of hemorrhagic complication and the highest anti-Xa activity under extracorporeal support or the mean heparin dose.

### Statistical analysis

Variables are presented as median [interquartile, 25th–75th percentile] for non-normally distributed data. Categorical variables are presented as number (percentage). Quantitative variables were compared with a Mann–Whitney test, as appropriate. Qualitative variables were compared by the Fisher’s exact test or the Chi-square test. The primary outcome was bleeding event under ECMO. A Cox proportional hazard model was used to identify variables associated with bleeding. Variables entered in the model were non-collinear factors associated (*p* < 0.1) with the outcome of interest in univariate analysis. A threshold value of mean anti-Xa activity associated with hemorrhage during ECMO was identified by ROC curve analysis. The cohort was then dichotomized into two groups according to the threshold value of anti-Xa activity with the highest sensitivity and specificity, and a survival analysis was performed using the Kaplan–Meier method. Unadjusted tests were carried out for secondary outcomes. Pearson’s correlation analysis was performed to determine the correlation between mean anti-Xa and mean dose of heparin under ECMO. Significance was set to *p* < 0.05. Statistical analyses were performed using MedCalc Software (version 19.4).

## Results

**Fig. 1:**
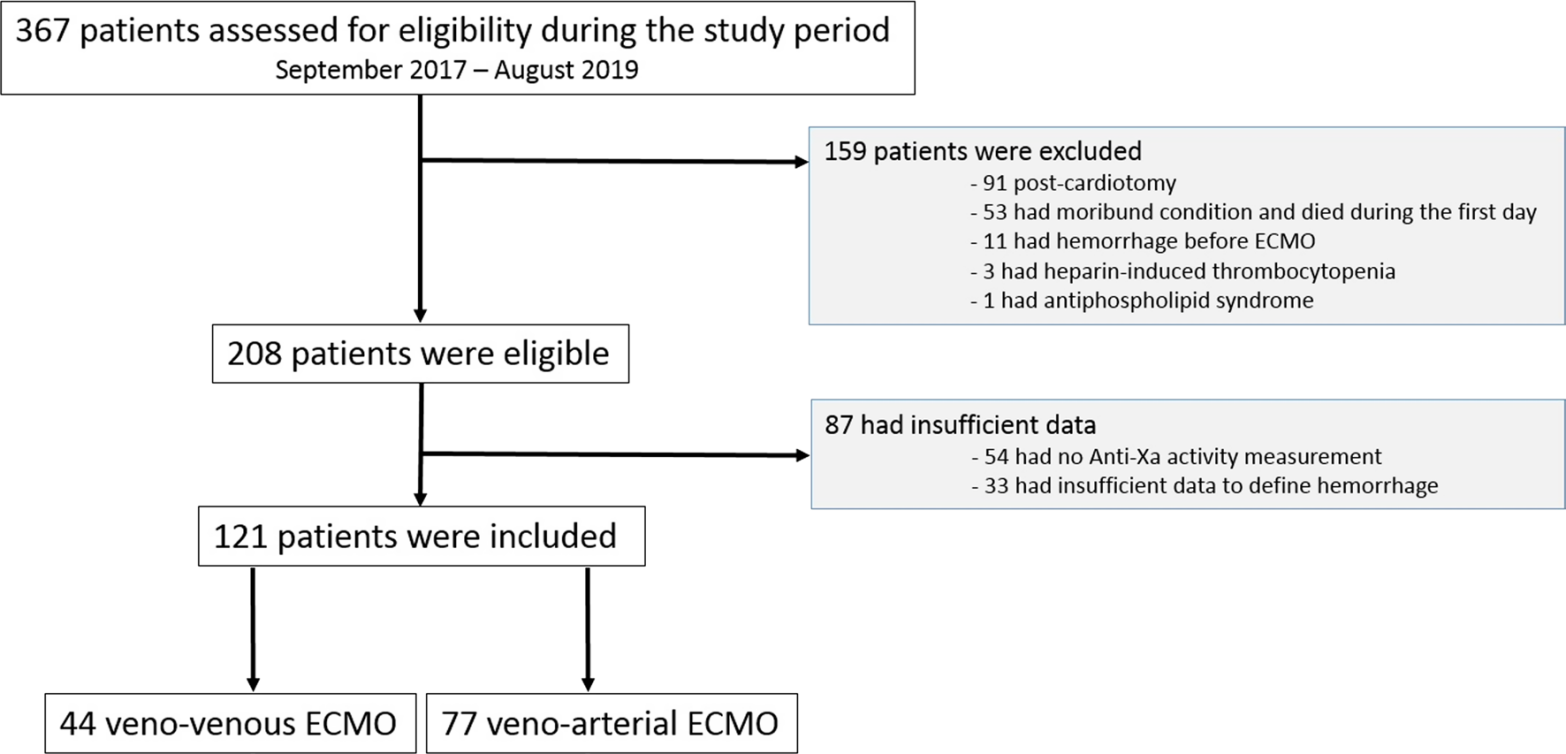
Flowchart of the study

Over the two years of the study period, 367 patients who were treated by an ECMO were assessed for eligibility (see Additional file 2). Among these 367 screened patients, 159 presented exclusion criteria, and 87 charts presented insufficient data. One hundred and twenty-one patients were included in the final analysis (Fig. 1 and Additional file 3). Of these, 35 (29%) presented a hemorrhagic complication.

Baseline characteristics of the study population are described in Table 1 and did not differ between groups of patients with or without bleeding event except for SAPS II, which was higher in the bleeding group (68 [44–82] versus 56 [40–70], *p* = 0.02). Characteristics of ECMO run (Table 2) were also similar among groups except for the duration of ECMO support (8 [4–13] days in the bleeding group versus 5 [3–9] in the non-bleeding group (*p* = 0.03)). Focusing on associated circulatory support, intra-aortic balloon pump (IABP) or IMPELLA were present in 43% of patients in the bleeding group and 16% in the non-bleeding group (*p* = 0.08), irrespective of the type of ECMO. In the subgroup of the 77 ECLS, an IABP or IMPELLA device was present in 52% of patients in the bleeding group versus 25% of patients in the group free from hemorrhage (*p* = 0.03). Specific characteristics of ECMO type are described in Additional file 4.

**Table 1:** Baseline characteristics of the study population, according to the occurrence of hemorrhage or not

	All patients (*n* = 121)	Bleeding group (*n* = 35)	Non-bleeding group (*n* = 86)	*p* value
Demographics				
Age (years)	51 [39–61]	50 [45–63]	51 [35–61]	0.5
Weight (kg)	80 [70–91]	80 [70–94]	77 [70–80]	0.4
BMI (kg/m^2^)	26.0 [22.9–31.0]	26.0 [22.5–31.0]	26.0 [22.9–31.0]	0.9
Male gender, *n* (%)	80 (67)	26 (74)	54 (63)	0.3
Comorbidity				
Hypertension, *n* (%)	30 (25)	11 (31)	19 (22)	0.4
Ischemic cardiomyopathy, *n* (%)	38 (31)	12 (34)	26 (30)	0.9
Dilated cardiomyopathy, *n* (%)	16 (13)	3 (9)	13 (15)	0.4
Atrial fibrillation, *n* (%)	9 (7)	1 (3)	8 (9)	-
Chronic limb ischemia, *n* (%)	3 (2)	2 (6)	1 (3)	0.9
Active smoking, *n* (%)	41 (34)	14 (40)	26 (30)	0.4
Asthma, *n* (%)	7 (6)	2 (6)	5 (6)	-
COPD, *n* (%)	5 (4)	3 (9)	2 (2)	-
Obstructive sleep apnea, *n* (%)	6 (5)	1 (3)	5 (6)	-
Type II diabetes, *n* (%)	11 (9)	3 (9)	8 (9)	0.9
Immunosuppression, *n* (%)	9 (7)	3 (9)	6 (7)	-
Active cancer, *n* (%)	7 (6)	3 (9)	4 (3)	-
Hematologic malignancy, *n* (%)	6 (5)	2 (6)	4 (5)	-
Alcohol consumption, *n* (%)	21 (17)	7 (20)	14 (16)	0.6
Dysthyroidism, *n* (%)	6 (5)	3 (9)	3 (3)	-
Long-term medication				
Antiplatelet agents, *n* (%)	12 (10)	5 (14)	7 (8)	0.3
Oral anticoagulants, *n* (%)	6 (5)	1 (3)	5 (6)	0.7
ICU admission				
SAPS II	59 [42–74]	68 [44–82]	56 [40–70]	0.02
SOFA score	10 [7–13]	11 [7–13]	10 [7–12]	0.8
Ventilation				
Tidal volume (mL/kg)	5.6 [4.3–6.2]	5.0 [3.9–5.9]	5.7 [4.3–6.4]	0.3
PEEP (cmH_2_O)	10 [6–12]	10 [7–12]	10 [6–12]	0.5

Data reported as number (percentage) or median [Interquartile range]

Tidal volume in milliliters of ideal body weight

BMI, body mass index, COPD, chronic obstructive pulmonary disease, PEEP, positive end-expiratory pressure, SAPS, simplified acute physiology score, SOFA, sequential organ failure assessment

**Table 2:** ECMO run characteristics

	All patients (*n* = 121)	Bleeding group (*n* = 35)	Non-bleeding group (*n* = 86)	*p* value
Indication, *n* (%)				0.8
Cardiogenic shock	47 (39)	13 (37)	34 (39)	
Refractory cardiac arrest	18 (15)	6 (17)	12 (14)	
ARDS	41 (34)	13 (37)	28 (33)	
Intoxication	15 (12)	3 (9)	12 (14)	
Type				0.4
Veno-venous	44 (36)	14 (40)	30 (35)	
Veno-arterial	77 (64)	21 (60)	56 (65)	
Cannula insertion				0.4
Femoro-femoral	74 (61)	21 (60)	53 (61)	
Femoro-jugular	41 (34)	14 (40)	27 (31)	
Subclavian	4 (3)	0 (0)	4 (5)	
AVALON dual-lumen catheter^a^	2 (2)	0 (0)	2 (3)	
Implantation day				
SOFA score	11 [8–13]	11 [8–13]	12 [8–14]	0.6
Glasgow coma scale^b^	14 [3–15]	14 [3–15]	14 [3–15]	0.8
Hemoglobin (g/dL)	11.5 [9.8–14.1]	11.8 [9.9–14.0]	11.2 [9.2–14.2]	0.7
Prothrombin ratio (%)	73 [60–87]	74 [58–91]	72 [60–86]	0.7
Platelets (G/mm^3^)	230 [170–303]	233 [199–309]	223 [160–288]	0.4
Creatininemia (µmol/L)	106 [79–154]	106 [79–148]	109 [82–160]	0.5
During ECMO support				
Maximum creatininemia (µmol/L)	153 [97–255]	160 [110–224]	145 [89–267]	0.4
Maximum bilirubinemia (µmol/L)	20 [13–35]	24 [14–32]	20 [12–38]	0.9
Associated IABP / IMPELLA^b^, *n* (%)	29 (24)	15 (43)	14 (16)	0.004
Associated RRT, *n* (%)	40 (33)	12 (34)	28 (33)	0.9
Length of ECMO support (days)	6 [3–10]	8 [4–13]	5 [3–9]	0.03

Data reported as number (percentage) or median [interquartile range]

ARDS, acute respiratory distress syndrome; ECMO, extracorporeal membrane oxygenation; IABP, intra-aortic balloon pump; SOFA, sequential organ failure assessment; RRT, renal replacement therapy

^a^AVALON Elite Bi-Caval Dual Lumen Catheter, Getinge, Sweden

^b^IMPELLA, Abiomed Europe, Germany

**Fig. 2:**
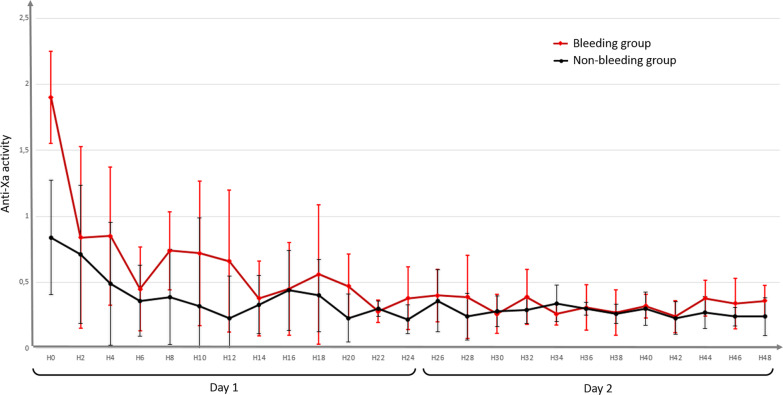
Two hourly mean anti-Xa activity evolution during the first two days. Precisely timed data on anti-Xa levels was only available during the first 48 h. The bars represent standard deviations. *p* = 0.04 by repeated measures regression analysis

Hemostatic data are described in Table 3, and evolution of mean anti-Xa activity within the first two days is shown in Fig. 2. In the univariate analysis, mean anti-Xa activity was significantly higher in the bleeding group than in the non-bleeding group (0.38 [0.29–0.67] versus 0.33 [0.22–0.42] IU/mL, respectively, *p* = 0.01). The maximum anti-Xa was also higher in the bleeding group than in the non-bleeding group (0.83 [0.47–1.46] versus 0.66 [0.36–0.91] IU/mL, respectively, *p* = 0.05). There was no significant difference regarding the averaged heparin dosage (11 [7–16] IU/kg/h in the bleeding group versus 12 [8–15] IU/kg/h in the non-bleeding group, *p* = 0.9). When focusing on the subgroup of patients with VV-ECMO versus VA-ECMO, no difference was observed regarding bleeding events (14 (32%) in the VV-ECMO group versus 21 (27%) in the VA-ECMO group, *p* = 0.2) or mean anti-Xa activities (0.33 [0.18–0.44] IU/mL in the VV-ECMO group versus 0.36 [0.25–0.47], *p* = 0.08), but maximal anti-Xa activity was significantly higher in the VA-ECMO group (0.75 [0.51–1.05] IU/mL) than in the VV-ECMO group (0.49 [0.24–0.79] IU/mL, *p* = 0.002).

**Table 3:** Hemostatic data for the two groups

	Bleeding group (*n* = 35)	Non-bleeding group (*n* = 86)	*p* value
Heparin doses			
Mean dose (IU/kg/h)	11 [7–16]	12 [8–15]	0.9
Dose at the occurrence of bleeding (IU/kg/h)	13 [7–16]	–	–
Anti-Xa (IU/mL)			
Mean anti-Xa during ECMO support	0.38 [0.29–0.67]	0.33 [0.22–0.42]	0.01
Maximum anti-Xa	0.83 [0.47–1.46]	0.66 [0.36–0.91]	0.05
Anti-Xa at the occurrence of bleeding	0.33 [0.15–0.50]	–	–
Platelet function			
Minimum platelets during ECMO (/mm^3^)	93 [57–162]	91 [60–120]	0.6
Platelets at the occurrence of bleeding (/mm^3^)	109 [63–178]	–	–
Antiplatelet agents during ECMO, *n* (%)	24 (68)	59 (69)	0.9
Hemorrhagic complications			
Needing 2 packed RBC or more / drop of hemoglobin of 2 g/dL or more	23 (66)	–	–
Characterized by its location	12 (34)	–	–
Needing specific intervention	11 (31)	–	–
Delay between ECMO and hemorrhage (days)	3 [2–6]	–	–
Type of bleeding, *n* (%)			
Intracerebral hemorrhage	9 (26)	–	–
Bleeding on cannula site	14 (40)	–	–
Oropharyngeal bleeding	7 (20)	–	–
Pericardial bleeding	3 (9)	–	–
Pulmonary hemorrhage	2 (6)	–	–
Hemothorax	2 (6)	–	–
Gastrointestinal bleeding	1 (3)	–	–
Hematuria	1 (3)	–	–
Massive metrorrhagia	1 (3)	–	–
Multiple-site bleeding	7 (20)	–	–
Bleeding leading to death, *n* (%)	8 (23)	–	–
Number of packed RBC during ECMO	7 [4–13]	2 [0–5]	< 0.0001
Thrombotic complications, *n* (%)	8 (23)	20 (23)	0.7
Ischemic stroke	4 (11)	6 (7)	
Membrane oxygenation replacement needed	2 (8)	4 (5)	
Limb ischemia	0 (0)	3 (3)	
Deep vein thrombosis	3 (9)	8 (9)	
30-day mortality, *n* (%)	17 (48)	20 (24)	0.009

Data reported as count (percentage) or median [interquartile range]

ECMO, extracorporeal membrane oxygenation; RBC, red blood cells

TE complications occurred in 23% of patients in both groups. Similarly, regarding VV- or VA-ECMO specifically, there was no difference between groups for hemorrhagic or TE events (see Additional file 5). When assessing specifically TE events, mean anti-Xa was 0.29 [0.18–0.37] in the TE group and 0.36 [0.25–0.46] in the group without TE event (*p* = 0.048). Overall 30-day mortality was 39% and was significantly higher in the bleeding group than in the non-bleeding group (48% versus 24%, respectively, *p* = 0.009).

Multivariate analysis of variables associated with bleeding event under ECMO is presented in Table 4. Factors associated with hemorrhage were mean anti-Xa activity (*p* = 0.0001) and SAPS II (*p* = 0.02) after adjusting for cofounding variables (center and associated mechanical support).

**Table 4:** Multivariable Cox proportional hazard model of factors associated with bleeding under ECMO

	HR	95% CI	*p* value
Center	0.83	0.65–1.03	0.09
SAPS II	1.02	1.01–1.05	0.02
Mean anti-Xa	16.10	3.94–65.82	0.0001
Associated IMPELLA/IABP	0.89	0.54–1.47	0.6

CI, confidence interval; HR, hazard ratio; SAPS, simplified acute physiology score; IABP, intra-aortic balloon pump

**Fig. 3:**
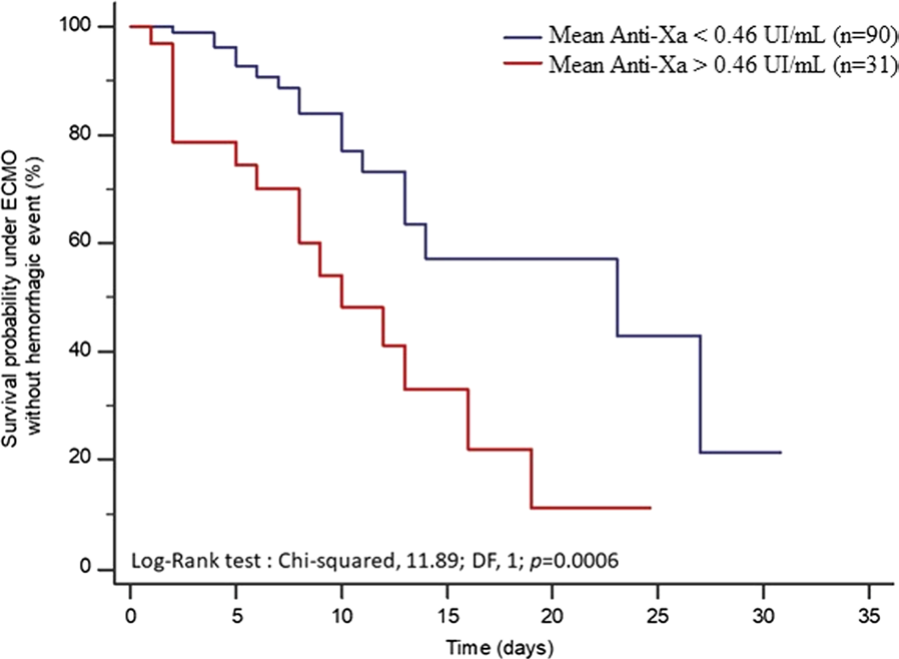
Kaplan–Meier curve showing the probability of survival without a bleeding event under ECMO depending on anti-Xa value. The cutoff of 0.46 IU/mL is the value with the best sensibility/specificity to predict the occurrence of a hemorrhagic event (Sensibility: 46%, Specificity: 87%, AUROC: 0.65; 95%CI, 0.58–0.73; *p* = 0.018)

By ROC curve analysis, a threshold value of a mean anti-Xa of 0.46 IU/mL was identified as predictor of hemorrhagic event (Sensibility: 46%; Specificity: 87%; area under the ROC (AUROC): 0.65; 95%CI, 0.58–0.73; *p* = 0.018; C-index, 0.74; 95%CI, 0.65–0.81 meaning an acceptable discriminating capacity of the model to predict a hemorrhagic event). As shown in Fig. 3, with this cutoff value, the probability of survival under ECMO without hemorrhagic event was significantly lower when mean anti-Xa was superior to 0.46 IU/mL (*p* = 0.0006). Outcomes by ECMO subtypes are shown in Additional file 6.

There was no correlation between mean anti-Xa value and heparin dose under ECMO: Pearson's r value 0.190 (− 0.011–0.376), *p* = 0.064 (Additional file 7).

## Discussion

In this multicenter cohort study assessing hemorrhagic events under VV- and VA-ECMO, we found a significant association between the anti-Xa activity and the risk of bleeding. A cutoff value of 0.46 IU/mL for mean anti-Xa activity under ECMO was identified to predict hemorrhagic events with acceptable accuracy. These findings may be of importance in a clinical point of view since anticoagulation treatment is one of the few modifiable risk factors of bleeding under ECMO. Moreover, the association between anti-Xa monitoring and bleeding under ECMO is interesting in routine clinical practice and could lead to reviewing of current anticoagulation targets.

To date, this is the second study focusing on a relationship between anti-Xa activity and hemorrhagic events under ECMO. A first study by Stulak et al. compared aPTT and anti-Xa to heparin doses and found no significant association between maximum anti-Xa and bleeding events. [21]. Other studies found a significant relationship between anticoagulation parameters and hemorrhage or transfusion, but most of these studies concerned aPTT [8, 19, 20]. However, anti-Xa activity seems to be more suitable in ECMO patients [16, 21, 25].

Regarding extrinsic validity of our study, our population does not differ from the ECMO population in the literature. We used a well-established definition for hemorrhage [15] to fit with other bleeding events evaluations. The population of our study had a bleeding rate of 29% under ECMO, which is comparable with other studies [3, 5, 26]. We chose to exclude post-cardiotomy patients because we believe that this specific population should be studied separately for the following reasons. First, the probability of bleeding in this population is particularly high without the use of ECMO because of the hemodilution during surgery and the presence of a surgical wound and drains. The anticoagulation targets may differ in this population, inducing increased anticoagulation requirements in case of mechanical valve to no anticoagulation in case of overt bleeding in the postoperative period. In addition, depending on the duration of the cardiopulmonary bypass, the risk of hemolysis with increased plasma-free hemoglobin is higher and may lead to less accurate anti-Xa values. The mortality in this population is known to be higher under ECMO when compared to non ECMO patients, thus the added mortality is concerning.

The meaning and implications of the results of this study are multiple. Based on the significantly higher mean anti-Xa value in the bleeding group, we hypothesize that anticoagulation goals may be revised. In VV-ECMO patients, the mean anti-Xa activity was in the range from 0.2 to 0.4 IU/mL, recommended in this setting [17], and lower than the critical value of 0.46 IU/mL observed in our population. However, the threshold of anti-Xa activity could be lower in VV-ECMO. Herman et al. evaluated the absence of heparin therapy in a population of thrombocytopenic patients with hematologic malignancies and concluded that this method was safe [27]. Carter et al. confirmed the safety of a heparin-sparing anticoagulation strategy, especially for polytraumatized patients with contraindication to anticoagulation [28]. Kurihara et al. showed less transfusion rates without circuit thrombosis nor a difference regarding mortality in a group of patients with low levels of heparin [29]. Finally, in the EOLIA trial [3], the anticoagulation regimen was low in the ECMO group with aPTT goals of 40 to 55 s or anti-Xa activity between 0.2 and 0.3 IU/mL. Only 2% of massive bleeds and 2% of hemorrhagic strokes were reported, a rate lower than the usual incidence of intracranial hemorrhages observed under ECMO [30].

Concerning VA-ECMO, anti-Xa targets are usually between 0.3 and 0.7 IU/mL [15], which we consider high in view of our results. Unfortunately, the literature is poor regarding systemic anticoagulation regimen in nonsurgical VA-ECMO patients. One bias is the acute disease-induced need of VA-ECMO, including dilated cardiomyopathies with thrombosis risk , ischemic cardiomyopathies with initial indication for anticoagulation and antiplatelet agents, and pathophysiologic patterns increasing the risk of atrial fibrillation. Considering that VA-ECMO patients accounted to two third of the cohort, we consider that our results provide an important contribution for reevaluation of the threshold of anti-Xa in this setting. However, for both VV and VA ECMO, downgrading anticoagulation targets should be done with caution, as the mean anti-Xa was significantly lower in patients with TE events.

We systematically gave a bolus of heparin at the initiation of extracorporeal cannulation. Maximal anti-Xa measures were more frequent in the first hours after ECMO initiation. Despite that, we failed to identify the maximal anti-Xa level as an independent risk factor of bleeding. This result could be explained by the low number of assays available during the first hours of ECMO when cannulation was done by the mobile ECMO team. However, the observed signal could question the relevance of the recommended bolus of heparin [15]. Heparin-coated cannulas and biocompatibility of new membranes should allow for a short period without anticoagulation. As the bolus is meant to prevent thrombosis, which was not the primary outcome measure evaluated in this study, other studies are needed to evaluate its benefits/risks balance. At the moment, some teams do not use heparin in the first hours of ECMO, without observing thrombosis [31].

There are important limitations to consider in the interpretation of our findings. First, it is a retrospective study, with all the biases that this implies. In particular, we had missing data, such as pre-ECMO platelet counts and coagulation parameters, partly due to the organization in two centers (Lille and Rouen) with a mobile ECMO team, cannulating in peripheral centers and bringing patients into ECMO centers. Other missing data can be of importance, such as plasma free hemoglobin or hyperbilirubinemia. These parameters were not systematically dosed at the time of the anti-Xa measurement and can be result confounders, lowering the value of anti-Xa activity [32]. Second, the main judgment criterion, namely the mean anti-Xa activity, does not fit a real-life interpretation. This value can be influenced by the length of ECMO. Shorter ECMO durations could have overestimated the mean anti-Xa activity in the hemorrhagic group, because the highest measures correspond to the beginning of ECMO support, which can be notably influenced by the heparin bolus. An interesting parameter to record would have been the prescribed target of anti-Xa to calculate the observed-to-prescribed ratio of anti-Xa activity. This information was, however, not reported in all medical records. Third, our observations do not take into account the mechanistic parameters of hemostasis, and new technologies such as thromboelastography (TEG) should be evaluated in this context [19, 33]. Fourth, the use of varying anti-Xa goals between the centers and the study populations could introduce selection bias, but it also introduces variability of anticoagulation intensity within the population, which improve the ability of the study to determine the association between anticoagulation intensity and bleeding. Finally, with a median ECMO support duration of 6 [3–10] days, this short-term support cohort may not be generalizable to centers practicing long-term ECMO runs for patients waiting for their heart or lung transplant.

Conversely, the major strengths come from our case-mix population in our multicentric model and from the size of our cohort with a large number of patients. This study included both VV- and VA-ECMO. Most of the studies focus only on VV- [3, 6, 7] or VA-ECMO [31]. ECMO devices are composed of pumps, membranes, and heparin-coated cannulas that does not differ between VV- and VA-ECMO, both with similar flows. We thought that separating the two techniques for a specific evaluation of heparin-induced bleeding was not necessary. This aspect seems to be confirmed, as no difference was observed for hemorrhagic complications among VV- and VA-ECMO in our study, highlighting that bleeding may be more favored by the anticoagulation than the technique itself. Recent ECMO studies focusing on the hemostatic specificities of ECMO also did not separate the two techniques [5, 33, 34].

## Conclusion

In this retrospective multicenter study evaluating a VV- and VA-ECMO population, mean anti-Xa was identified as an independent risk factor for hemorrhagic complications, with a critical cutoff value of 0.46 IU/mL. Our results prompt to investigate the question of anticoagulation targets prospectively. ECMO being increasingly utilized for rescue therapy and for which the optimal anticoagulation strategy is still uncertain, these results are part of an emerging literature that agrees toward a restrictive anticoagulation regimen that could be beneficial for outcome in these severe patients.

## Supplementary Information

**Additional file 1.** Analyzers and reagents used to measure Anti-Xa in the three centers.

**Additional file 2: Figure 1.** Number of ECMO implemented by month in the three centers during the study period

**Additional file 3: Figure:** Flowchart of the study detailed by center

**Additional file 4: Table:** ECMO characteristics detailed by type.

**Additional file 5: Figure:** Hemorrhagic and thrombotic events depending on ECMO type.

**Additional file 6: Table:** ECMO outcomes detailed by type.

**Additional file 7.** Correlation between mean anti-Xa and mean heparin dose under ECMO.

## Data Availability

The datasets used and/or analyzed during the current study are available from the corresponding author on reasonable request.

## Abbreviations

aPTT: Activated partial thromboplastin time
ACT: Activated clotting time
ARDS: Acute respiratory distress syndrome
BMI: Body mass index
CKD: Chronic kidney disease
COPD: Chronic obstructive pulmonary disease
ECCOR: Extracorporeal CO2 removal
ECLS: Extracorporeal life support
ECMO: Extracorporeal membrane oxygenation
ELSO: Extracorporeal life support organization
HIT: Heparin-induced thrombocytopenia
ICU: Intensive care unit
IU: International units
mL: Milliliter
OHCA: Out of hospital cardiac arrest
PR: Prothrombin ratio
RBC: Red blood cells
RRT: Renal replacement therapy
SAPS: Simplified acute physiology score
SOFA: Sequential organ failure assessment
TEG: Thromboelastography
TE: Thrombo-embolic
VA: Veno-arterial
VV: Veno-venous
vWF: Von Willebrand factor
