# Supplementary material for: Anti-Xa activity and hemorrhagic events under extracorporeal membrane oxygenation (ECMO): a multicenter cohort study
Source: Crit Care. 2021 Apr 2;25:127. doi: 10.1186/s13054-021-03554-0 (PMC8019180; doi:10.1186/s13054-021-03554-0)
Supplement: Supplementary file 4 — Additional file 4: Table: ECMO characteristics detailed by type. [file 13054_2021_3554_MOESM4_ESM.docx]

**Additional file 4**

**Anti-Xa activity and Hemorrhagic events under Extracorporeal Membrane Oxygenation (ECMO): A multicenter cohort study**

Richard DESCAMPS, MD^1^, Mouhamed D. MOUSSA, MD^2^, Emmanuel BESNIER, MD, PhD^3^, Marc-Olivier FISCHER, MD, PhD^4^, Sébastien PREAU, MD, PhD^5^, Fabienne TAMION, MD, PhD^6^, Cédric DAUBIN, MD^1^, Nicolas COUSIN, MD^5^, André VINCENTELLI, MD, PhD^7^, Julien GOUTAY, MD^5^, Damien DU CHEYRON, MD, PhD^1^

1. Department of Medical Intensive Care, Caen University Hospital, F-14000, Caen, France

2. Univ. Lille, Inserm, CHU Lille, Surgical Critical Care, Department of Anesthesiology and Critical Care, Institut Pasteur de Lille, UMR1011-EGID, 59000, Lille, France

3. Department of Anesthesiology and Critical Care, Rouen University Hospital, F-76000, Rouen, France

4. Department of Anesthesiology and Critical care, Caen University Hospital, F-14000 Caen, France.

5. Department of Medical Intensive Care, Lille University Hospital, F-59000, Lille, France

6. Normandie Univ, UNIROUEN, Inserm U1096, FHU- REMOD-VHF, 76000 Rouen, France and Department of Medical Intensive Care, Rouen University Hospital, F-76000, Rouen, France

7. Univ. Lille, Inserm, CHU Lille, Department of Cardiac Surgery, Institut Pasteur de Lille, UMR1011-EGID, 59000, Lille, France

**Corresponding author:**

Richard DESCAMPS

Mail: descamps-r@chu-caen.fr

Phone number: +33231064708

Postal address: Service de Médecine Intensive-Réanimation, Centre Hospitalier Universitaire de Caen, Avenue de la côte de Nacre, 14033 Caen, France.

**Additional file 4 - table:** ECMO characteristics detailed by type.

|  | | All patients | | Bleeding group | | Non-bleeding group | *p* value |
| --- | --- | --- | --- | --- | --- | --- | --- |
| Veno-venous, n (%) | 44 | | 14 (32) | | 30 (68) | |  |
| Indication |  | |  | |  | |  |
| ARDS | 37 (84) | | 11 (79) | | 26 (87) | | 0.7 |
| Asthma | 3 (7) | | 1 (7) | | 2 (7) | | - |
| Tracheal injury | 4 (9) | | 2 (14) | | 2 (7) | | - |
| Vascular access |  | |  | |  | |  |
| Femoro-jugular | 41 (93) | | 14 (100) | | 27 (90) | | 0.5 |
| Jugular only (AVALON) | 2 (5) | | 0 | | 2 (7) | | - |
| Femoro-femoral | 1 (2) | | 0 | | 1 (3) | | - |
| Veno-arterial, n (%) | 77 | | 21 (27) | | 56 (72) | |  |
| Indication |  | |  | |  | |  |
| Refractory cardiac arrest | 18 (23) | | 6 (29) | | 12 (21) | | 0.2 |
| Ischemic cardiomyopathy | 22 (29) | | 6 (29) | | 16 (29) | | - |
| Myocarditis / Dilated cardiomyopathy | 25 (32) | | 7 (33) | | 18 (32) | | - |
| Intoxication | 9 (12) | | 0 | | 9 (16) | | - |
| Refractory ventricular tachycardia | 2 (3) | | 1 (5) | | 1 (2) | | - |
| Pulmonary embolism | 1 (1) | | 1 (5) | | 0 | | - |
| Vascular access |  | |  | |  | |  |
| Femoro-femoral | 73 (95) | | 21 (100) | | 52 (93) | | 0.6 |
| Femoro-axillary | 4 (5) | | 0 | | 4 (7) | | - |
| Associated circulatory support | 25 (32) | | 11 (52) | | 14 (25) | | 0,03 |
| IABP | 15 (20) | | 8 (38) | | 7 (13) | | 0,4 |
| IMPELLA | 10 (13) | | 3 (14) | | 7 (13) | | 0,9 |

ARDS: Acute Respiratory Distress Syndrome, IABP: Intra-Aortic Balloon Pump.
