# Supplementary material for: Anti-Xa activity and hemorrhagic events under extracorporeal membrane oxygenation (ECMO): a multicenter cohort study
Source: Crit Care. 2021 Apr 2;25:127. doi: 10.1186/s13054-021-03554-0 (PMC8019180; doi:10.1186/s13054-021-03554-0)

**Additional file 5**

**Anti-Xa activity and Hemorrhagic events under Extracorporeal Membrane Oxygenation (ECMO): A multicenter cohort study**

Richard DESCAMPS, MD^1^, Mouhamed D. MOUSSA, MD^2^, Emmanuel BESNIER, MD, PhD^3^, Marc-Olivier FISCHER, MD, PhD^4^, Sébastien PREAU, MD, PhD^5^, Fabienne TAMION, MD, PhD^6^, Cédric DAUBIN, MD^1^, Nicolas COUSIN, MD^5^, André VINCENTELLI, MD, PhD^7^, Julien GOUTAY, MD^5^, Damien DU CHEYRON, MD, PhD^1^

1. Department of Medical Intensive Care, Caen University Hospital, F-14000, Caen, France

2. Univ. Lille, Inserm, CHU Lille, Surgical Critical Care, Department of Anesthesiology and Critical Care, Institut Pasteur de Lille, UMR1011-EGID, 59000, Lille, France

3. Department of Anesthesiology and Critical Care, Rouen University Hospital, F-76000, Rouen, France

4. Department of Anesthesiology and Critical care, Caen University Hospital, F-14000 Caen, France.

5. Department of Medical Intensive Care, Lille University Hospital, F-59000, Lille, France

6. Normandie Univ, UNIROUEN, Inserm U1096, FHU- REMOD-VHF, 76000 Rouen, France and Department of Medical Intensive Care, Rouen University Hospital, F-76000, Rouen, France

7. Univ. Lille, Inserm, CHU Lille, Department of Cardiac Surgery, Institut Pasteur de Lille, UMR1011-EGID, 59000, Lille, France

**Corresponding author:**

Richard DESCAMPS

Mail: descamps-r@chu-caen.fr

Phone number: +33231064708

Postal address: Service de Médecine Intensive-Réanimation, Centre Hospitalier Universitaire de Caen, Avenue de la côte de Nacre, 14033 Caen, France.

**Additional file 5 - figure:** Hemorrhagic and thrombotic events depending on ECMO type.


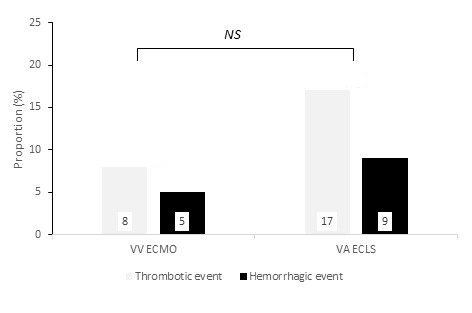

Supplement: Supplementary file 5 — Additional file 5: Figure: Hemorrhagic and thrombotic events depending on ECMO type. [file 13054_2021_3554_MOESM5_ESM.docx]
