# Supplementary material for: Anti-Xa activity and hemorrhagic events under extracorporeal membrane oxygenation (ECMO): a multicenter cohort study
Source: Crit Care. 2021 Apr 2;25:127. doi: 10.1186/s13054-021-03554-0 (PMC8019180; doi:10.1186/s13054-021-03554-0)
Supplement: Supplementary file 6 — Additional file 6: Table: ECMO outcomes detailed by type. [file 13054_2021_3554_MOESM6_ESM.docx]

**Additional file 6**

**Anti-Xa activity and Hemorrhagic events under Extracorporeal Membrane Oxygenation (ECMO): A multicenter cohort study**

Richard DESCAMPS, MD^1^, Mouhamed D. MOUSSA, MD^2^, Emmanuel BESNIER, MD, PhD^3^, Marc-Olivier FISCHER, MD, PhD^4^, Sébastien PREAU, MD, PhD^5^, Fabienne TAMION, MD, PhD^6^, Cédric DAUBIN, MD^1^, Nicolas COUSIN, MD^5^, André VINCENTELLI, MD, PhD^7^, Julien GOUTAY, MD^5^, Damien DU CHEYRON, MD, PhD^1^

1. Department of Medical Intensive Care, Caen University Hospital, F-14000, Caen, France

2. Univ. Lille, Inserm, CHU Lille, Surgical Critical Care, Department of Anesthesiology and Critical Care, Institut Pasteur de Lille, UMR1011-EGID, 59000, Lille, France

3. Department of Anesthesiology and Critical Care, Rouen University Hospital, F-76000, Rouen, France

4. Department of Anesthesiology and Critical care, Caen University Hospital, F-14000 Caen, France.

5. Department of Medical Intensive Care, Lille University Hospital, F-59000, Lille, France

6. Normandie Univ, UNIROUEN, Inserm U1096, FHU- REMOD-VHF, 76000 Rouen, France and Department of Medical Intensive Care, Rouen University Hospital, F-76000, Rouen, France

7. Univ. Lille, Inserm, CHU Lille, Department of Cardiac Surgery, Institut Pasteur de Lille, UMR1011-EGID, 59000, Lille, France

**Corresponding author:**

Richard DESCAMPS

Mail: descamps-r@chu-caen.fr

Phone number: +33231064708

Postal address: Service de Médecine Intensive-Réanimation, Centre Hospitalier Universitaire de Caen, Avenue de la côte de Nacre, 14033 Caen, France.

**Additional file 6 – table:** ECMO outcomes detailed by type.

|  | | All patients | Bleeding group | | Non-bleeding group |
| --- | --- | --- | --- | --- | --- |
| Veno-venous | 44 (100) | | 14 (32) | 30 (68) | |
| Death under ECMO | 10 (23) | | 4 (29) | 6 (20) | |
| Weaning | 34 (77) | | 10 (71) | 24 (80) | |
| 30-Day Mortality | 16 (36) | | 6 (42) | 10 (33) | |
| Veno-arterial | 77 (100) | | 21 (27) | 56 (72) | |
| Death under ECMO | 19 (25) | | 7 (33) | 12 (21) | |
| Weaning | 41 (53) | | 9 (43) | 32 (57) | |
| Cardiac transplantation | 9 (12) | | 3 (14) | 6 (11) | |
| Long-term Mechanical circulatory support | 3 (4) | | 0 (0) | 3 (4) | |
| LVAD (Heartmate, Thoratec, USA) | 2 (3) | | 0 (0) | 2 (3) | |
| BiVAD (Syncardia, USA) | 1 (1) | | 0 (0) | 1 (1) | |
| Multi-organ donation | 2 (3) | | 1 (5) | 1 (2) | |
| 30-Day Mortality | 31 (41)* | | 11 (52) | 20 (36)* | |

Values represented as number (percentage). LVAD = Left Ventricular Assist Device, BiVAD = Biventricular Assist Device. *missing data.
